# Polymeric Forms of Plant Flavonoids Obtained by Enzymatic Reactions

**DOI:** 10.3390/molecules27123702

**Published:** 2022-06-09

**Authors:** Malgorzata Latos-Brozio, Anna Masek, Małgorzata Piotrowska

**Affiliations:** 1 Institute of Polymer and Dye Technology, Faculty of Chemistry, Lodz University of Technology, Stefanowskiego 16, 90-537 Lodz, Poland; anna.masek@p.lodz.pl; 2 Institute of Fermentation Technology and Microbiology, Faculty of Biotechnology and Food Sciences, Lodz University of Technology, Wólczańska 71/173, 90-530 Lodz, Poland; malgorzata.piotrowska@p.lodz.pl

**Keywords:** naringenin, enzymatic polymerization, laccase, horseradish peroxidase

## Abstract

Naringenin is one of the flavonoids originating from citrus fruit. This polyphenol is mainly found in grapefruit, orange and lemon. The antioxidant and antimicrobial properties of flavonoids depend on their structure, including the polymeric form. The aim of this research was to achieve enzymatic polymerization of naringenin and to study the properties of poly(naringenin). The polymerization was performed by methods using two different enzymes, i.e., laccase and horseradish peroxidase (HRP). According to the literature data, naringenin had not been polymerized previously using the enzymatic polymerization method. Therefore, obtaining polymeric naringenin by reaction with enzymes is a scientific novelty. The research methodology included analysis of the structure of poly(naringenin) by NMR, GPC, FTIR and UV-Vis and its morphology by SEM, as well as analysis of its properties, i.e., thermal stability (DSC and TGA), antioxidant activity (ABTS, DPPH, FRAP and CUPRAC) and antimicrobial properties. Naringenin oligomers were obtained as a result of polymerization with two types of enzymes. The polymeric forms of naringenin were more resistant to thermo-oxidation; the final oxidation temperature T_o_ of naringenin catalyzed by laccase (poly(naringenin)-laccase) was 28.2 °C higher, and poly(naringenin)-HRP 23.6 °C higher than that of the basic flavonoid. Additionally, due to the higher molar mass and associated increase in OH groups in the structure, naringenin catalyzed by laccase (poly(naringenin)-laccase) showed better activity for scavenging ABTS^+•^ radicals than naringenin catalyzed by HRP (poly(naringenin)-HRP) and naringenin. In addition, poly(naringenin)-laccase at a concentration of 5 mg/mL exhibited better microbial activity against *E. coli* than monomeric naringenin.

## 1. Introduction

Phytochemicals, including polyphenols, are valued because of their excellent antioxidant properties and other biological activities [1,2]. Flavonoids belonging to the polyphenol group deserve special attention. These compounds are widely distributed in the plant kingdom. They consist of a benzene A ring, condensed with oxygenated heterocyclic C ring and phenyl B ring attached at carbon C2. The biological activities of a large number of flavonoids have been described and are generally considered to be beneficial for health [3,4]. An important activity of flavonoid compounds is their antiradical ability, which can provide protection against various types of free radicals, such as hydroxyl, superoxide, peroxide and lipid-peroxyl radicals [3,4].

The structure of polyphenols has a significant impact on their antioxidant capacity. The configuration, number and distribution of functional groups influence the free radical reaction mechanism and the activity of phenolic compounds. The antiradical ability of flavonoids depends on their capacity to resist the oxidizing action of reactive free radicals [5,6]. The most important structural features of the flavonoids that provide effective antiradical activity are an *o*-dihydroxyl group in the B ring, a C2=C3 double bond linked to a C4=O group on the C ring, and hydroxyl groups in the C3 and C5 positions [7,8]. Moreover, literature data indicate that the polymeric structure of flavonoids can exhibit stronger antiradical properties and higher antimicrobial activity [8] and also better thermal stability [9]. The example of tannins shows that procyanidin dimers and trimers were more effective than monomeric flavonoids against superoxide anions; however, the dimer and trimer activity differed little. In contrast, tetramers showed greater anti-oxidation activity through peroxynitrite- and superoxide- than trimers, whereas heptamers and hexamers had significantly better superoxide scavenging properties than trimers and tetramers. It has been found that possibly the increasing degree of polymerization to some extent increases the effectiveness of the procyanidins against various radicals. The extensive conjugation between 3-OH and B-ring catechol groups, together with abundant β_4 → 8_ linkages, has given polymeric tannins radical scavenging properties by increasing the stability of its radical [8]. However, the relationship between the polymeric structure of flavonoids and their antioxidant and pharmacological properties is poorly studied [8,9,10].

The following possibilities for flavonoid polymerization have been described in literature: enzymatic polymerization [11,12,13,14], photopolymerization [15], and self-condensation of flavonoids [16,17], as well as polymerization with a cross-linking compound [18,19]. Despite the existence of scientific publications on the polymerization of many flavonoids by various methods, the polymerization of naringenin is very poorly studied. Only the polymerization of naringenin with the cross-linking agent glycerol diglycide ether (GDE) is known [20]. Naringenin (4,5,7-trihydroxy-flavanone) is a flavanone from the group of flavonoids found in large amounts in citrus fruits. The antioxidant properties and pharmacological activity (anti-inflammatory, neuroprotective, anti-cancer and anti-diabetic) of naringenin have been characterized [21,22].

The beneficial properties of this flavanone depend on the arrangement of correlated functional groups in its structure. The 5,7-*m*-dihydroxy system in ring A is responsible for stabilizing the naringenin structure after donating electrons to free radicals. The hydroxyl (OH) groups show significant reactivity towards reactive oxygen species (ROS) as well as to reactive nitrogen species. Due to the combination of 5-OH and 4-oxo substituents, naringenin has the ability to chelate heavy metals [3,8].

As a result of polymerization with glycerol diglycide ether (GDE), a strongly cross-linked poly(naringenin) structure was obtained. The polymeric form of naringenin had greater activity in reducing ABTS^+•^ (2,2′-azinobis-(3-ethylbenzothiazoline-6-sulfonate)) and DPPH^•^ (2,2-diphenyl-1-picrylhydrazyl) free radicals than the monomer. The introduction of additional OH groups to the cross-linked naringenin could contribute to the improvement of antioxidant properties measured by ABTS and DPPH tests. Moreover, naringenin, after polymerization, had antimicrobial activity against *Candida albicans*, whereas the monomer did not show activity against this yeast [20].

Among the data on flavonoid polymerization, enzymatic reaction is the most widely described method. The polymeric flavonoid structures were obtained by enzymatic polymerization with the use of two groups of enzymes: laccase and peroxidase. Polymeric forms of catechin [11], rutin [23,24], quercetin [25] and esculin [14] were obtained with the use of the enzyme laccase. Another enzyme utilized in the polymerization of flavonoids is horseradish peroxidase (HRP). As a result of the reaction with horseradish peroxidase, the polymeric structures of catechin [12,13,26] and quercetin [27] were synthesized. The enzymatic reactions of polyphenols influenced their biological activity. The antioxidant activity of poly(flavonoids) may depend on the molecular weight of the compounds as well as the type and position of the bonds (Mw, PDI, C-C or C-O bridges). Chebil et al. showed a decrease in the antiradical activity of poly(rutin) with an increase in its molecular weight Mw [14]. Moreover, the polymeric catechin obtained in the enzymatic reaction had a stronger inhibitory effect on DPPH^•^ free radicals. Utilizing xanthine oxidase inhibition assay, it was found that enzymatic polymerization of polyphenols (rutin, esculin, catechin and epigallocatechin gallate) increased their antioxidant capacity [14,23,24].

The aim of this research was to achieve enzymatic polymerization of naringenin and to study the relationship between the structure obtained and its antioxidant properties, thermal stability and antimicrobial activity. The polymerization was performed by two methods with the use of different enzymes, i.e., laccase and horseradish peroxidase (HRP). Naringenin had not previously been polymerized using the enzymatic polymerization method. Obtaining polymeric naringenin by reaction with enzymes and studying the relationship between its structure and properties is a scientific novelty of this research.

## 2. Results and Discussion

### 2.1. Analysis of the Structure of Poly(naringenin) Obtained by Reaction with the Enzymes Laccase and Horseradish Peroxidase (HRP)

#### 2.1.1. FTIR and UV-Vis Spectroscopy

**Figure 1:**
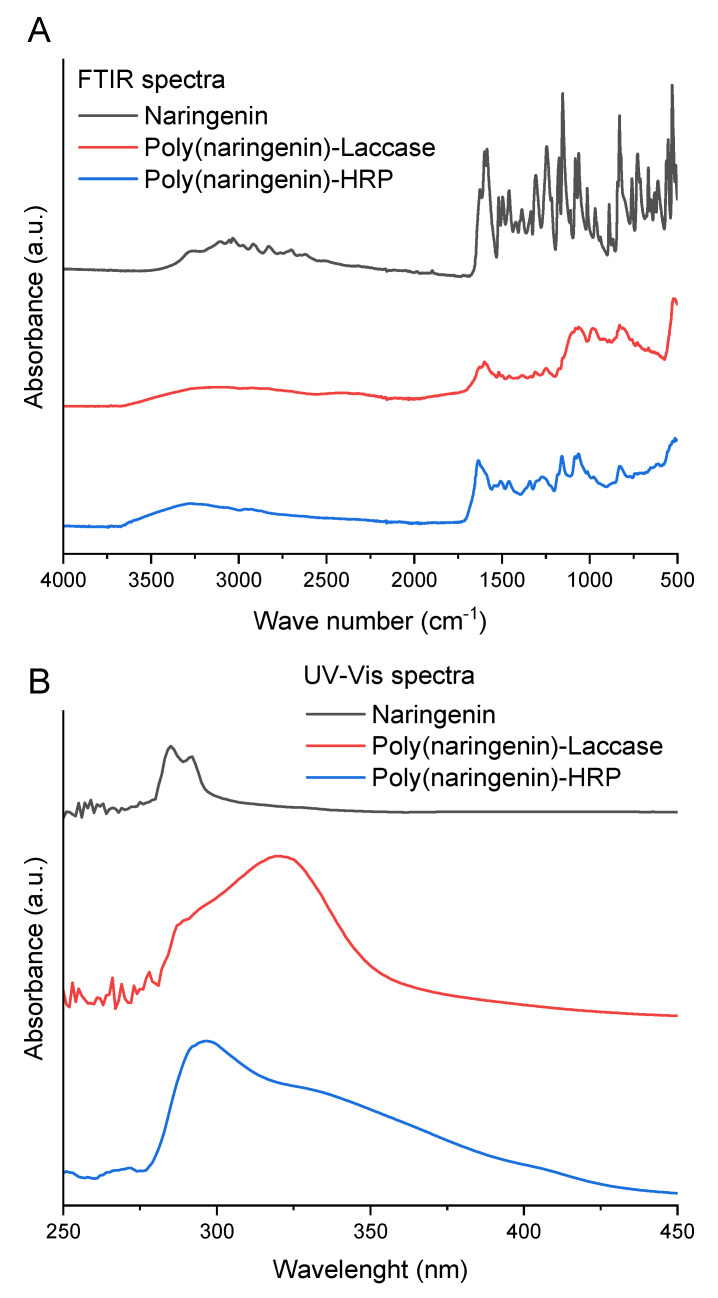
(**A**) FTIR spectroscopy and (**B**) UV-Vis spectroscopy of naringenin, poly(naringenin)-laccase and poly(naringenin)-HRP.

Infrared FTIR and UV-Vis spectroscopy allowed for an initial analysis of the polymeric structure of the polymers produced. Figure 1 shows the FTIR (Figure 1A) and UV-Vis (Figure 1B) spectra of reference naringenin and naringenin obtained by enzymatic polymerization with laccase and HRP. Polymeric forms of flavonoids have specific and characteristic functional groups. According to the literature data, the polymeric form of naringenin, obtained in reaction with glycerol diglycidyl ether GDE has specific bands, such as wide bands at 3700–3000 cm^−^^1^ corresponding to the formation of free OH, and 1370–1250 cm^−1^ aryl stretching vibrations, as well as bands at 1061 cm^−1^ corresponding to C-CO-C in ketones [20]. These specific bands were also visible on the FTIR poly(naringenin)-laccase and poly(naringenin)-HRP spectra. The wide band in the 3700–2500 cm^−1^ range related to changed OH groups, the characteristic aryl stretching vibrations (1363–1210 cm^−1^ poly(naringenin)-laccase and 1366–1205 cm^−1^ poly(naringenin)-HRP), and C-CO-C groups in ketones (1058 cm^−1^ poly(naringenin)-laccase and 1063 cm^−1^ in poly(naringenin)-HRP spectra) were observed. Clear changes in the band area corresponding to OH groups may suggest that hydroxyl groups were involved in the naringenin polymerization process. The polymeric naringenin contains many individual units of this flavonoid. Thus, the overall polymeric naringenin may have more of all OH groups than a single naringenin unit, which can be seen in the FTIR spectrum. The peaks at 831 cm^−1^ (poly(naringenin)-laccase) and 827 cm^−1^ (poly(naringenin)-HRP), as well as the peak at 980 cm^−1^ (poly(naringenin)-laccase), may also suggest the formation of new C-O-C bonds [28].

Figure 1B illustrates the UV-Vis spectra of naringenin and its polymeric forms. The standard naringenin had two specific peaks at 287 and 292 nm, whereas both polymeric forms of naringenin were distinguished by wide peaks in the 280–450 nm range. According to the literature, the peak between 200–300 nm and a broad band between 300 and 550 nm are characteristic of the powder of polymeric forms of naringenin obtained in the cross-linking reaction with GDE [20]. Analogous changes in the UV-Vis spectra were also observed for rutin oligomers [24]. The UV-Vis spectrum of rutin showed two absorption maxima at 282 and 359 nm corresponding to the π-π* transition of aromatic electrons. For the oligomeric rutin, the 359 nm band was much larger and showed a hypsochromic shift, suggesting the involvement of the rutin B ring in oligomer formation.

#### 2.1.2. Nuclear Magnetic Resonance (NMR) Analysis

The next step was the analysis of the poly(naringenin)-laccase and poly(naringenin)-HRP structure by NMR. Unfortunately, due to the incomplete solubility of the polymerized samples in DMSO, the ^1^H NMR spectra were not of perfect quality and the measurements may be burdened with some imperfection.

**Figure 2:**
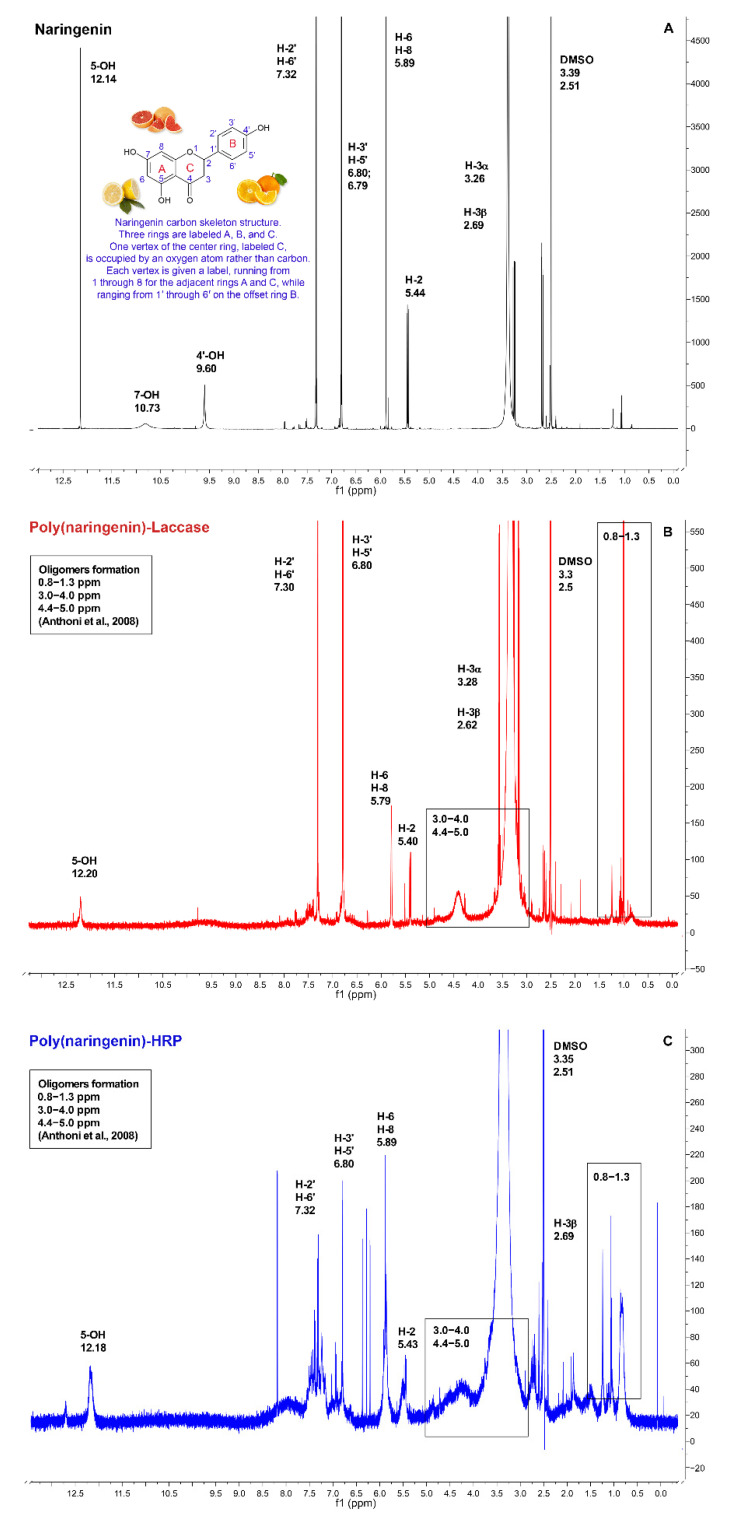
^1^H NMR of naringenin (**A**), poly(naringenin)-laccase (**B**) and poly(naringenin)-HRP (**C**), d6-DMSO, 250 MHz. According to Anthoni et al. [24] peaks in the ranges of 0.8–1.3 ppm, 3.0–4.0 ppm and 4.4–5.0 ppm were characteristic for the oligomeric formation of flavonoid.

The study began with the NMR spectrum of the reference naringenin (Figure 2A). The monomeric naringenin had shifts characteristic of hydroxyl groups (5-OH, 7-OH, 4′-OH) as well as for aromatic compounds. The shifts shown in Figure 2A were consistent with the ^1^H NMR spectra literature data for naringenin [29]. As a result of enzymatic polymerization with laccase (Figure 2B) and HRP (Figure 2C), there was a disappearance or other changes in shifts corresponding to the hydroxyl groups (5-OH, 7-OH and 4′-OH). This suggests that the polymerization process occurred with the participation of hydroxyl groups and the formation of C-O bonds. The participation of hydroxyl groups in the polymerization is also evidenced by the changes observed in the FTIR spectra (Figure 1A) in the area of bands corresponding to the hydroxyl groups. Moreover, it was shown in the scientific literature that oligomeric rutin had specific peaks in the ^1^H NMR spectra in the ranges 0.8–1.3 ppm, 3.0–4.0 ppm and 4.4–5.0 ppm [24]. These peaks, corresponding to the oligomers, were also found in the poly(naringenin)-laccase and poly(naringenin)-HRP spectra. Characteristic chemical shifts for oligomeric forms of flavonoids also suggest that C-C bonds were involved in the polymerization reaction.

**Figure 3:**
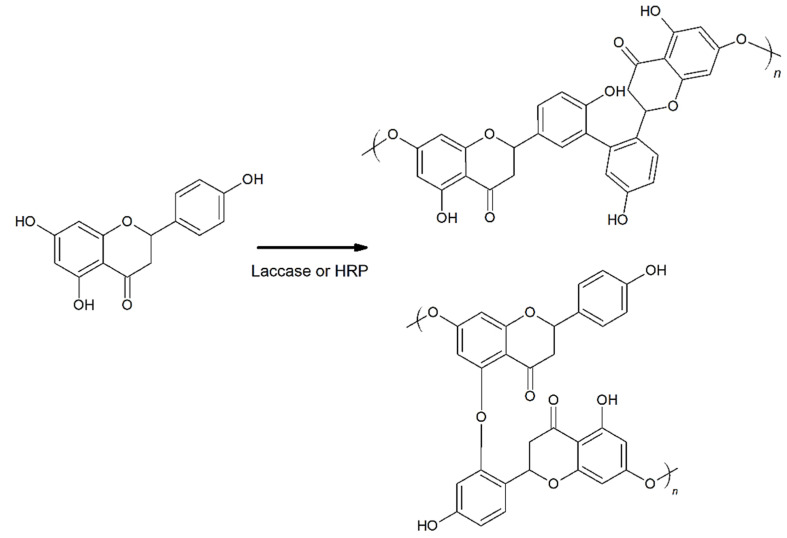
Proposed pathways of naringenin enzymatic polymerization with laccase or HRP, where n–number of mers in poly(naringenin).

A possible scheme for the enzymatic polymerization of naringenin is proposed in Figure 3. Lacasse and HRP enzymes have been used to polymerize several polyphenols. According to the manufacturer, Laccase EC 1.10.3.2, a glycoprotein, is an extracellular multicopper enzyme and is considered as a metal [30]. The enzyme is widely distributed in fungi and also found among the higher plants and bacteria, as well as insects. Laccase oxidizes aromatic and nonaromatic compounds. This enzyme is commonly used in industry for delignification, dye bleaching, paper processing, textile dye transformation and plant fibre modification, and also ethanol production [30]. Horseradish peroxidase is isolated from horseradish roots (*Amoracia rusticana*) and belongs to the ferroprotoporphyrin group of peroxidases. Enzyme HRP is a single chain polypeptide containing four disulfide bridges. It is used in biochemistry. HRP readily combines with hydrogen peroxide, and the resultant HRP-H_2_O_2_ complex can oxidize a wide variety of hydrogen donors [31].

The mechanisms of enzymatic polymerization of catechin with laccase and HPR have been well described by Hosny & Rosazza [13]. Horseradish peroxidase (HRP; EC 1.11.1.7) catalyzed the H_2_O_2_ dependent oxidative coupling (+)-catechin to form three different C-C biphenyl dimers. The second enzyme, laccase Rhus vernicifera, catalyzed the formation of two new adducts of catechin and hydroquinone. Typically, (+)-catechin polymers were formed by couplings between the ring A of one unit and the ring B of the other unit in a so-called “head to tail” polymerization process. 8-hydroxy-(+)-catechin and catechin dimers (dehydrodicatechins) were obtained by H_2_O_2_-dependent peroxidase, and also chemical oxidation of (+)-catechin under alkaline conditions. Structurally, dehydrodicatechins differ from one another by the biphenyl or phenyl ether (C-C or C-O-C) types of linkage, the position of the interflavan linkage, as well as the relative conformations of biphenyl or biphenyl ether moieties [13].

Moreover, Bruno and co-authors [27] explained the mechanism of the quercetin polymerization reaction with HRP. According to these authors, the reaction with HRP involved the initial two-electron oxidation of the ferric enzyme to form an oxidized intermediate (HRP-I) by adding hydrogen peroxide. Subsequently, the quercetin monomer was oxidized by the intermediate (HRP-I) to form a monomeric radical. The radical species joined together to form dimers first, then trimers, which was repeated until an oligomer of considerable size was obtained. These oligomeric forms differed from the well-known natural anthocyanins [27].

The mechanism of enzymatic polymerization of naringenin was analogous to the reactions of other polyphenols. Based on NMR and FTIR analysis, it was found that hydroxyl groups in the 5-OH, 7-OH and 4′-OH position may play a key role in the polymerization reaction. Moreover, the formation of naringenin oligomers may be accompanied by novel C-O-C and C-C bonds. Unfortunately, the limited solubility of the polymeric forms of naringenin significantly hinders the precise analysis of its structure and polymerization path.

#### 2.1.3. Gel Permeation Chromatography (GPC)

**Figure 4:**
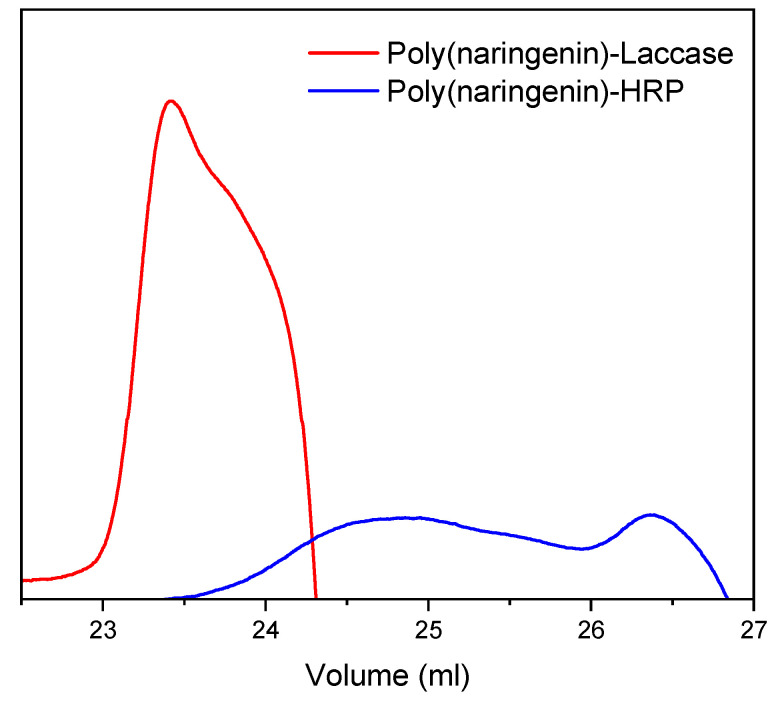
Gel permeation chromatography GPC traces of poly(naringenin)-laccase and poly(naringenin)-HRP samples.

GPC chromatography allowed determination of the molar mass of the polymeric forms of flavonoids. The results are summarized in Table 1 and Figure 4.

Naringenin is a low molecular weight compound with a molecular weight of 272.25 g/mol. For this reason, the flavonoid sample was below the measuring range of GPC chromatography. The molar mass of poly(naringenin)-HRP was 9.972 × 10^2^ g/mol. Based on a comparison of the molecular weight of naringenin and the polymeric form of naringenin, it was found that poly(naringenin)-HRP could be a tetramer. Poly(naringenin) obtained in the reaction with laccase was characterized by a higher molecular weight (4.584 × 10^3^ g/mol). Given that the polymer is a chain of at least 10,000 or more units of monomer [32], poly(naringenin)-laccase was an oligomer and contained approximately 17 units. Nevertheless, the thermal analysis of the samples described later in the manuscript showed that poly(naringenin)-laccase had a glass transition temperature T_g_ characteristic of polymeric forms. The polydispersity index PDI (M_w_/M_n_) describes the heterogeneity of the molar mass of the polymer. The PDI for poly(naringenin)-laccase was equal to 1.040 [-], and for poly(naringenin)-HRP 1.392 [-]. The monodisperse (homogeneous) polymers have a PDI value of 1. An example of such polymers are biopolymers [32]. The PDI index of poly(naringenin) -lacase indicated that it was a homogeneous (monodisperse) compound, which is typical for biopolymers. On the other hand, the poly(naringenin)-HRP sample was characterized by some heterogeneity of the molar mass, which could be caused by the polymerization conditions.

#### 2.1.4. Microscopic Analysis

**Figure 5:**
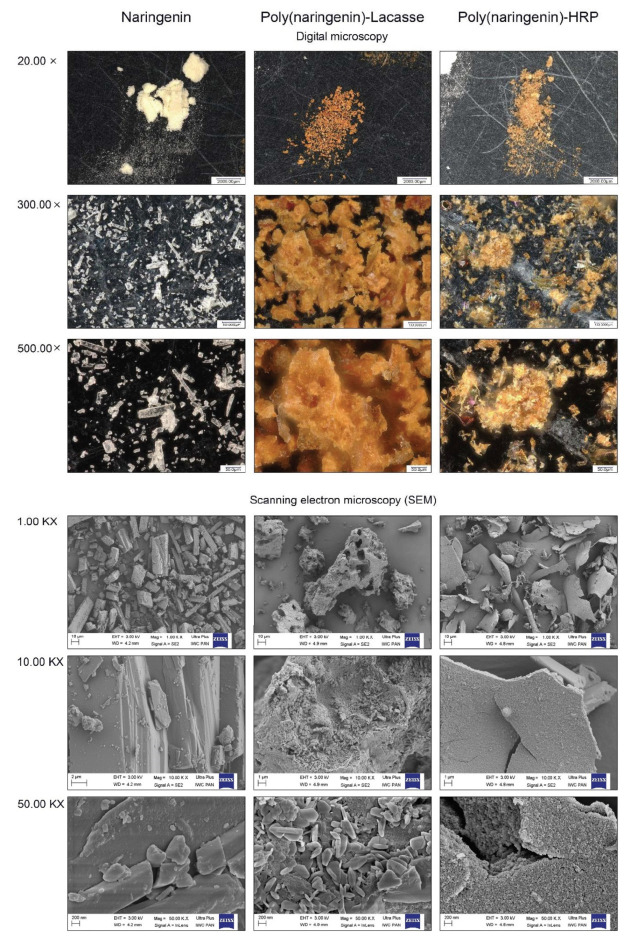
Digital images (20×, 300×, 500× magnification) and SEM images (1.00 kx, 10.00 kx and 50.00 kx magnification) of naringenin, poly(naringenin)-laccase and poly(naringenin)-HRP.

Figure 5 shows the digital images (at 20×, 300×, 500× magnification) and SEM images (at 1 kx, 10 kx and 50 kx magnification) of naringenin, poly(naringenin)-laccase and poly(naringenin)-HRP. The morphologies of the monomeric flavonoid and the polymeric forms obtained with different enzymes were completely different. Needle structures were clearly visible in the naringenin sample. The poly(naringenin) obtained with laccase resembled the shape of lumps (1 kx) in which small lamellar structures were visible at 10 kx and 50 kx magnification. The poly(naringenin)-HRP morphology was lamellar. In the sample at 50 kx magnification, the lamellas had a spongy structure. Based on the analysis of SEM images, it should be concluded that the polymerization method and the type of enzyme used have a significant impact on the morphology of poly(naringenin), while both polymeric forms contained lamellar structures in the samples, unlike the needle-shaped naringenin.

### 2.2. Analysis of the Properties of Poly(naringenin) Obtained by Reaction with the Enzymes Laccase and Horseradish Peroxidase (HRP)

#### 2.2.1. Antioxidant Capacity

Table 2 shows the results of the determination of the activity of naringenin and poly(naringenin) to scavenge ABTS^+•^ and DPPH^•^ free radicals, as well as the ability of polyphenols to reduce iron (FRAP) and copper (CUPRAC) ions.

Unpolymerized naringenin at a concentration of 1 mg/mL showed the best activity for scavenging ABTS^+•^ radicals (55.0 ± 0.22%). This monomeric compound was characterized by a slight ability to inhibit DPPH^•^ radicals and iron ions, and a slightly better ability to reduce copper ions. The polymeric form of naringenin obtained with the HRP enzyme had weaker antioxidant activity than the monomer. The ABTS^+•^ free radical scavenging ability was 30.4 ± 0.16%. Poly(naringenin)-HRP showed little ability to inhibit ABTS^+•^ radicals and iron ions (less than naringenin), and also exhibited no activity to reduce copper ions. Poly(naringenin)-laccase had different properties. Unlike naringenin and poly(naringenin)-HRP, it presented a greater ability to inhibit ABTS^+•^ free radicals (85.7 ± 0.28%), but on the other hand, it did not show any activity to capture DPPH^•^ radicals. Poly(naringenin)-laccase, similarly to poly(naringenin)-HRP, was characterized by a lower ability to reduce transition metal ions (FRAP and CUPRAC methods) than the monomer. Overall, the polymeric forms of naringenin showed weaker antioxidant activity than the monomer (excluding the ABTS score for poly(naringenin)-laccase). These poorer results of the polymeric forms and the inconclusive trend in the results may be related to the limited solubility of the samples, as well as the use of different solvents for naringenin (EtOH) and polyflavonoid forms (water). Moreover, the poly(naringenin) solutions had a clearly yellow color, which could also adversely affect the results of spectrophotometric colorimetric tests.

In the DPPH, FRAP and CUPRAC tests (as well as in the ABTS assay for poly(naringenin)-HRP), lower antioxidant activity may be related to the fact that active OH groups (responsible for oxidation reactions) took part in polymerization reactions and served as links between monomers. Moreover, in naringenin, the linkage between 5-OH and 4-oxo [33] contributes to the chelation of compounds such as heavy metals. For poly(naringenin) obtained by cross-linking with GDE [20], it was found that the OH groups in the 5 and 4′ positions can participate in the cross-linking reaction and act as a link between monomers; therefore, the activity measured by the CUPRAC method may be lower than that of the monomer. In turn, the activity of naringenin and poly(naringenin) against iron ions was minimal [20], similar to this work.

Particularly noteworthy are the ABTS results for poly(naringenin)-laccase and poly(naringenin)-HRP, which are divergent from those for monomeric naringenin, i.e., poly(naringenin)-laccase had a greater degree of ABTS^+•^ radical inhibition, while poly(naringenin))-HRP had a lower degree of ABTS inhibition. These results may indicate that the polymerization with different enzymes proceeds according to different mechanisms, and the reaction products can have different structures and other properties, e.g., related to the oligomeric form, molar mass and C-C or C-O bridges. Poly(naringenin)-laccase had a higher molecular weight than poly(naringenin)-HRP as well as better activity against ABTS^+•^ radicals (compared with monomeric naringenin and poly(naringenin)-HRP). Poly(naringenin)-laccase with a higher molar mass may contain more OH groups in its structure that participate in the inhibition of free radicals. Moreover, the presence of more OH groups may change the nature of the compound to be more hydrophilic. The more hydrophilic nature of poly(naringenin)-laccase may result in better inhibition of ABTS^+•^ radicals than DPPH^•^ because the ABTS determination is intended for analysis of hydrophobic and hydrophilic antioxidants, whereas the DPPH method is dedicated to the examination of hydrophobic compounds.

Similar results regarding the ambiguity of the polymerization effect on the antioxidant properties of polyphenols are indicated in the literature. According to scientific reports, the source of the same enzyme may affect the antioxidant properties of the polyphenol obtained in the reaction with this enzyme. The polymeric rutin obtained in reaction with the laccase of *Pycnoporus coccineus*, *Pycnoporus sanguineus* or *Myceliophthora* had better ability to inhibit the AAPH radical compared with the monomer. On the other hand, poly(rutin), obtained in reaction with laccase from *Trametes versicolor*, had a lower DPPH^•^ radical scavenging effect than rutin. The lack of a clear tendency in the behavior of polymeric flavonoids was attributed to the various methods for determining the antioxidant activity, as well as the degree of polymerization [14]. In this article, the laccase obtained from *Aspergillus* sp. was used for the enzymatic polymerization of naringenin, and the polymer product obtained had better activity to reduce ABTS^+•^ radicals than the monomer.

The polymerization of naringenin with various enzymes, laccase and HRP, has shown that the structure and antioxidant properties of the final polymeric product depend on the type of enzyme used and the polymerization method.

#### 2.2.2. Thermal Analysis

**Figure 6:**
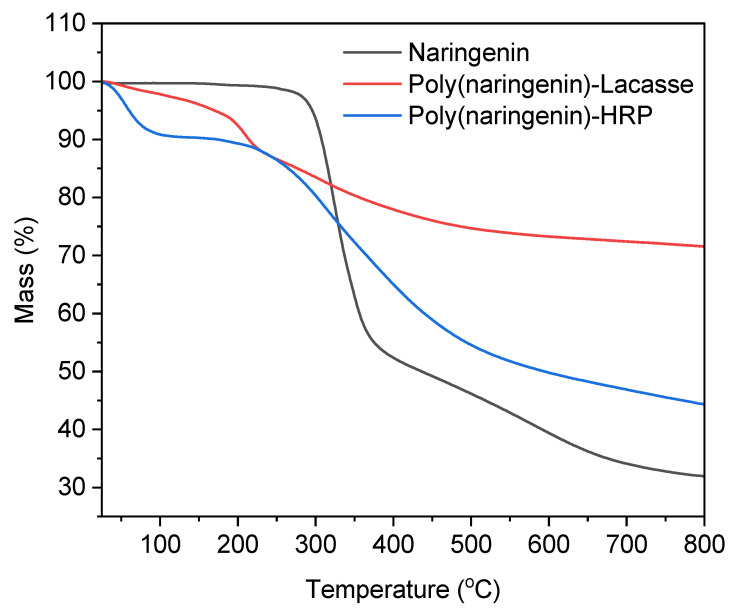
TG curves of naringenin and its polymeric forms obtained by an enzymatic reaction.

Figure 6 shows the thermogravimetric curves of naringenin and its polymerized forms. Table 3 summarizes the temperatures at which there was a weight loss of the samples, corresponding to 10% (T_10_), 20% (T_20_), 30% (T_30_), 50% (T_50_), 55% (T_55_) and 60% (T_60_).

The decomposition of monomeric naringenin was a one-step process. In the temperature range 290–380 °C, there was a 67% weight loss of the sample. Poly(naringenin)-laccase and poly(naringenin)-HRP had two stages of thermal degradation. The first stage of decomposition of poly(naringenin)-laccase occurred around 60 °C, and the weight loss was 5%. The second stage of decomposition was recorded in the temperature range of 100–550 °C. The second stage was accompanied by a weight loss in the sample amounting to 22%. For the poly(naringenin)-HRP sample, the first stage of decomposition also occurred around 60 °C, and the weight loss was 9%. The second stage of decomposition was found in the temperature range of 150–650 °C, and the corresponding weight loss of the sample was 45%. The multi-stage decomposition and the initial lower thermal resistance of the polymeric samples may be related to the decomposition of polymerization residues and the presence of process moisture but also to the gradual decomposition of the oligomeric fraction.

Based on the data summarized in Table 3, it was observed that the decomposition of the polymeric forms of naringenin began at lower temperatures than the decomposition of the reference sample (T_10_ of poly(naringenin)-laccase = 213 °C; T_10_ of poly(naringenin)-HRP = 173 °C; T_10_ of Naringenin = 307 °C). Particularly noteworthy is the final decomposition temperature of the polymeric naringenin. In the case of sample poly(naringenin)-laccase, the T_30_ value was higher by 463 °C than the T_30_ of naringenin, and for the poly(naringenin)-HRP, the T_55_ value was higher by 254 °C than the T_55_ of the monomeric form. Higher values of the final decomposition temperature of the polymeric naringenin indicate higher poly(naringenin)-laccase and poly(naringenin)-HRP thermal stability. Among the polymerized forms of flavonoids, poly(naringenin) obtained in reaction with laccase had the highest thermal stability; the total weight loss of the sample during the determination was only 27%, which proves that the material decomposed to a small extent under the given conditions. The poly(naringenin)-laccase sample had a higher molecular weight than poly(naringenin)-HRP. The better thermal stability of the polymeric forms of naringenin can be related to the cross-linking of the samples. Moreover, a higher molar mass of poly(naringenin)s can increase resistance to thermal degradation. The formation of new catechol-catechol bonds in the polymer backbone creates a thermally stable poly(flavonoid). Polymeric flavonoids with high thermal stability can, for example, be used as natural additives that can increase resistance to thermal degradation of environmentally friendly and biodegradable materials, including packaging materials.

**Figure 7:**
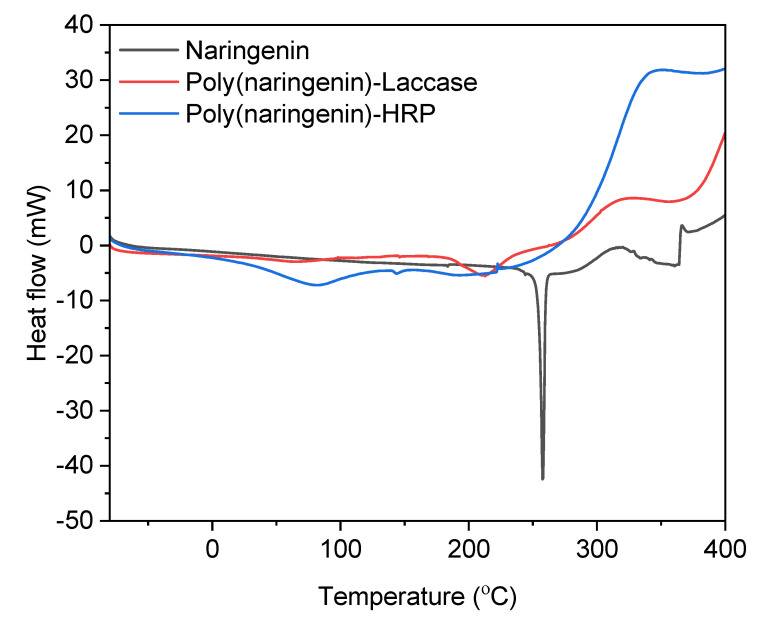
DSC analysis of reference naringenin and its polymeric forms.

As part of the thermal analysis, measurements were also made using the differential scanning calorimetry method. The results are shown in Figure 7 and in Table 4.

The DSC thermogram for poly(naringenin)-laccase showed a glass transition temperature of 66.6 °C. The polymeric forms of naringenin had higher initial and final oxidation temperatures: (poly(naringenin)-laccase—initial To by 13.7 °C and final by 28.2 °C; poly(naringenin)-HRP—initial T_o_ by 18.4 °C and final by 23.6 °C), and a higher enthalpy of oxidation: ΔH_o_, (poly(naringenin)-laccase about 2 times; poly(naringenin)-HRP about 7.7 times). The poly(naringenin)s obtained in the enzymatic reactions were, therefore, more resistant to oxidation. The polymeric naringenin (poly(naringenin)-laccase and poly(naringenin)-HRP) had a lower melting point than the monomeric flavonoid. This could be due to the presence of low molecular weight polymer fractions that could lower the T_m_.

Similar results for thermal analysis were demonstrated for the polymeric naringenin obtained by reaction with a cross-linking compound [20]. The thermogravimetric and differential scanning calorimetry analyses showed higher thermal stability of poly(naringenin) as well as increased resistance of the polymeric form of flavonoid to oxidation. The cross-linked structure of poly(naringenin) improved its thermal properties by limiting the access of heat and oxygen to the polymeric flavonoid particles, as well as slowing down its thermal decomposition and oxidation reactions.

#### 2.2.3. Antimicrobial Properties

Microorganisms can cause many clinical problems. *Escherichia coli* is one of the most-studied microorganisms worldwide. This bacterium is also an example of a model organism in microbiological and biotechnological research. *E.coli* is part of the physiological, saprophytic bacterial flora of the large intestine in warm-blooded animals and humans. In the intestine, it participates in the process of breaking down food residues and in the synthesis of vitamins B1, K, B12 and folic acid. It colonizes the skin, oral mucosa and the respiratory system. It becomes a pathogenic microorganism under appropriate favorable conditions. Commensal strains of E. coli in the presence of favorable factors can cause parenteral infections. Infections caused by E. coli are: food poisoning, urinary tract infections, neonatal meningitis (*E.coli K1*), organ abscesses, postoperative infections, sepsis (the most common Gram (-) rod causing sepsis), or inflammation of the bones, marrow and muscles. *E. coli* strains are also the most common etiological factor in nosocomial infections [34]. For studies of gram-positive bacteria, *Bacillus subtilis* is a model as important as *E. coli* for gram-negative bacteria. *B. subtilis* is the key gram-positive model bacterium for studies on physiology and metabolism. This bacterium is common, especially in soil, and also in spoiled food. It has been widely used as a cell factory for microbial production of chemicals, enzymes, and antimicrobial materials for industry, agriculture, and medicine [35]. Humans are also a natural reservoir of *Staphylococcus aureus*. The most common sites of colonization are skin, anterior nares, axilla, or perineum. *S. aureus* has been reported to cause a wide variety of infections, ranging from simple cutaneous abscesses to life-threatening fulminant necrotizing skin and soft tissue infections, septicemia, severe pneumonia, endocarditis, and toxic shock syndrome [36]. Fungi can also be a source of clinical problems. *Aspergillus niger* is one of the most common species of the genus *Aspergillus*, common in soil. It causes a disease called “black mold” in certain fruits and vegetables. *A. Niger* can cause a fungal infection of the ear (otomycosis) leading to lesions [37]. *Candida albicans* is another human fungal pathogen. *C. albicans* is often a benign member of the mucous flora; however, it can cause diseases of the mucous membranes with significant morbidity. It can cause life-threatening bloodstream infections in sensitive patients. An important feature of its biology is its ability to grow in the forms of yeasts, pseudo-hyphae and hyphae [38].

**Figure 8:**
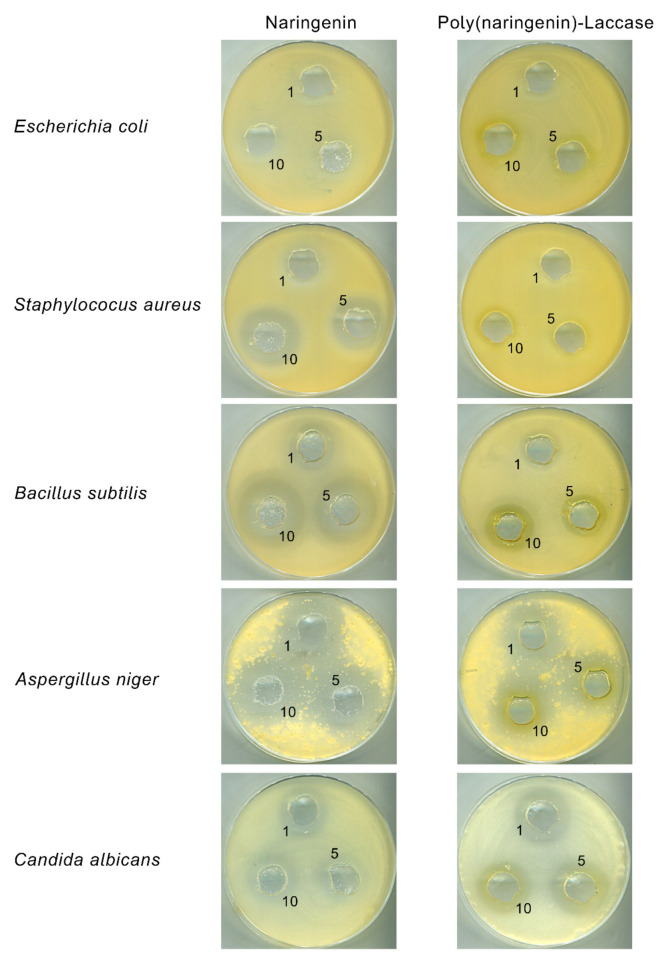
Photographic documentation of antimicrobial tests of naringenin and poly(naringenin)-laccase, where 1, 5, 10 means naringenin or poly(naringenin) concentration of 1 mg/mL, 5 mg/mL and 10 mg/mL.

Table 5 and Figure 8 present the results of the determination of antimicrobial activity of polyphenol samples after enzymatic polymerization with laccase. The tests were performed with the bacteria (*E. coli*, *S. aureus* and *B. subtilis*) and fungus (*A. niger* and *C. albicans*). Naringenin and poly(naringenin)-laccase showed a similar fungicidal effect against microorganisms *A. niger* and *C. albicans* (similar growth inhibition zone). Along with the increase in the concentration of polyphenols, there was a slight increase in the size of the growth inhibition zone of fungus.

Table 5 also summarizes the results of the microbiological tests for bacteria. Along with the increase in the concentration of naringenin and its polymeric forms, an increase in the growth inhibition zone of *B. subtilis* was also noted; however, the reference flavonoid had a larger zone of inhibition for the growth of this microorganism than poly(naringenin)-laccase. A similar, stronger antimicrobial effect of naringenin was also observed for *S. aureus* bacteria; naringenin showed a larger growth inhibition zone than poly(naringenin)-laccase. The polymeric form of naringenin showed better antimicrobial activity against *E. coli*. Naringenin in concentrations of 1 mg/mL and 5 mg/mL did not have growth inhibition zones for this bacterium, whereas the size of the zone for poly(naringenin)-laccase was 20 mm. For naringenin and poly(naringenin)-laccase at a concentration of 10 mg/mL, a 22 mm zone of was found for *E. coli*, which means that, at this concentration, the flavonoid and the poly(flavonoid) show similar antibacterial effects.

The antiviral/antibacterial properties of flavonoids correlate with certain structural elements of these compounds. For example, therapeutic activities against *E. coli* have been attributed mainly to the chemical structures in some formulations: methoxylation, glycosylation and hydroxylation. The number of hydroxyl groups is also an important factor. A greater number of hydroxyl groups reduces the hydrophobicity, which makes it difficult to break down flavonoids in biological membranes. However, some hydroxyl-rich flavonoids have been shown to be more active. Additional hydroxyl groups may reduce hydrophobicity, but due to higher C3 charges, the direct index of pharmacological activity may be higher. Therefore, the effect of hydrophobicity and electronic delocalization should be considered together for the attribution strength of hydroxylation [39].

Few scientific studies refer to the correlation between the structure of polymeric flavonoids and their antimicrobial activity. According to the literature [20], poly(naringenin) obtained as a result of polymerization with a cross-linking compound had better antimicrobial activity against *C. albicans* yeast then monomeric naringenin. According to the article, the lack of antimicrobial activity of poly(naringenin) with a cross-linking compound against *E. coli*, *S. aureus* and *B. subtilis* may be due to the low solubility of the samples, and thus difficulties in penetrating the microbial cells.

Poly(naringenin)-laccase activity against *E. Coli* was found to be better at concentrations of 1 and 5 mg/mL in the present work. The antimicrobial activity of poly(naringenin)-laccase against the remaining bacteria and fungi was similar or less than that of the reference naringenin, which may be due to the limited solubility of the poly(flavonoid) and the poorer availability of the slow-fused compound for microorganisms. Moreover, more hydroxyl groups can reduce the hydrophobicity and hinder the breakdown of poly(flavonoids) in biological membranes.

The scientific literature documents that the relationship between antioxidant activity and antimicrobial activity is correlated with the total polyphenol content in extracts of plant origin. Plant extracts with high antioxidant activity may show a significant antibacterial activity. Plant extracts containing the higher content of flavonoids and polyphenols may have a higher antioxidant and antimicrobial activity, which is the result of a higher content of active substances [40,41].

In the case of naringenin and poly(naringenin)-laccase, it was difficult to establish a clear correlation between antioxidant and antimicrobial properties. The polymeric form of naringenin showed greater antioxidant properties in the ABTS method, and poly(naringenin)-laccase at a concentration of 5 mg/mL exhibited better microbial activity against *E. coli* than monomeric naringenin. However, in the DPPH, FRAP and CUPRAC methods, the antioxidant activity and the ability to reduce metal ions by poly (naringenin) -laccase was lower than for the reference naringenin. Additionally, the antimicrobial activity of polymeric naringenin against *S. aureus*, *B. subtilis*, *A. niger* and *C. albicans* was similar or less than that of the naringenin. The properties of the oligomers could depend on the type of enzyme used, the polymerization method, and the method of analyzing antioxidant and antimicrobial properties. The results could also be influenced by the limited solubility of the polymeric forms resulting in some measurement error.

## 3. Materials and Methods

### 3.1. Polymerization of Naringenin with Laccase and Horseradish Peroxidase (HRP)

Reaction with laccase: 1 LAMU (Laccase Unit) is defined as the amount of enzyme which oxidizes 1 micromole of syringaldazine per minute at pH 7.5 and 30 °C. First, 6 mL of methanol (pure p.a., POCH) and 14 mL of phosphate buffer at pH 7.4 (1 M, Sigma-Aldrich, Saint Louis, MI, USA) were added to 0.2 g of naringenin (natural, 98%, MW: 272.25 g/mol, Sigma Aldrich). The solution was stirred at room temperature until the flavonoid was completely dissolved. 1500 units of laccase enzyme obtained from *Aspergillus* sp. (Sigma Aldrich) were then added to the solution. The solution was gently stirred with a magnetic stirrer for 64 h at room temperature and open to air. The reaction was accompanied by a color change of the solution from milky to beige. The solutions were then dialyzed in distilled water using dialysis membranes. After dialysis, the solutions were lyophilized to obtain the polymer.

Reaction with horseradish peroxidase (HRP): 10 mg of naringenin (natural, 98%, MW: 272.25 g/mol, Sigma Aldrich) was added to 6 mL of pH 7 phosphate buffer (0.1 M, CHEMPUR) and 4 mL of ethanol (96%, pure p.a., POCH). Then 4 mg of the enzyme HRP (type VI-A, Sigma Aldrich) was added to the solution with completely dissolved flavonoid. To start polymerization, 1.5 mL of 0.03% hydrogen peroxide (pure, CHEMPUR, Piekary Śląskie, Poland) was added to the solution in small portions with stirring. The reaction was carried out for 20 h with access to air, while gently stirring with a magnetic stirrer. As in the reaction with laccase, the solution was dialyzed and then lyophilized. The reaction was performed according to [27] with changes to the polymerization conditions appropriate for naringenin.

### 3.2. FTIR and UV-Vis Spectroscopy

Fourier transform infrared spectroscopy (FTIR) (A Nicolet 670 FTIR spectrophotometer, Thermo Fisher Scientific, Waltham, MA, USA) was used to examine the specific chemical groups of naringenin and poly(naringenin) powders. The examination of oscillating spectra in the wavenumber range from 4000 to 400 cm^−1^ allows definition of the functional groups with which the radiation interacted. The ATR accessory equipped with a single reflection diamond ATR crystal was applied in all the measurements.

The UV-Vis spectra of naringenin and poly(naringenin) at wavelengths of 190–1100 nm were recorded utilizing a UV-Vis spectrophotometer (Evolution 220, Thermo Fisher Scientific). The measurement was performed for samples with a concentration of 1 mg/mL dissolved in ethanol (naringenin) or in distilled water (polymeric naringenin) [9,20].

### 3.3. Nuclear Magnetic Resonance Characterization (NMR)

Nuclear magnetic resonance (^1^H NMR) was used to determine the naringenin conversion during the polymerization with enzymes. The ^1^H NMR spectra were recorded on a Bruker ADVANCE DPX 250 MHz instrument, with d6-DMSO as a solvent.

### 3.4. Gel Permeation Chromatography (GPC)

Gel permeation chromatography (GPC) was used to analyze the molecular weights and molecular weight distributions of the free polymers obtained by enzymatic polymerization of naringenin. The GPC measurements were performed utilizing a Wyatt instrument equipped with two Perfect Separation Solutions columns and one guard column (GRAM Linear (10 mµ, Mn between 800 Da–1,000,000)), light scattering, and deferential refractometer detectors (Wyatt). The measurements were carried out in DMF with 50 mmol LiBr as eluent at a flow rate of 1 mL/min. Polystyrene (PS) standards were used for calibration (PS from Mp = 1306 Da to Mp = 1,210,000 Da).

### 3.5. Microscopic Analysis

Digital microscopy: photos of naringenin and polymeric naringenin samples were obtained using a digital microscope VHX-7000 at magnifications of 20, 300 and 500 times.

Scanning electron microscopy (SEM): The morphology of the naringenin and poly(naringenin) powders was assessed on the basis of photos obtained with a scanning electron microscope (SEM) LEO 1530 (Carl Zeiss AG, Jena, Germany). The photos were taken at the following magnifications: 1000, 10,000 and 50,000×.

### 3.6. Antioxidant Activity Determined by ABTS, DPPH, FRAP and CUPRAC Methods

The antioxidant activity of monomeric and polymeric naringenin was determined by spectrophotometric methods based on the mechanism of hydrogen atom transfer (HAT) and single electron transfer (SET). ABTS and DPPH methods were applied, based on the reactions of quenching of synthetic free radicals ABTS^+•^ and DPPH^•^ [9,20,42].

Moreover, the ability of the compounds to reduce transition metal ions was investigated. The ability to reduce iron ions was determined by the FRAP method, and for copper ions by the CUPRAC method [9,20,42].

For the ABTS, DPPH, FRAP and CUPRAC tests, solutions of monomeric and polymeric naringenin at a concentration of 1.0 mg/mL were prepared. Naringenin is insoluble in water, but soluble in organic solvents such as, for example, alcohol [43]. Therefore, it was dissolved in ethanol for the determination of antioxidant activity. The polymeric naringenin was insoluble in ethanol but partially soluble in distilled water, so polymeric naringenin solutions were prepared with water.

The authors described the detailed methodology for ABTS, DPPH, FRAP and CUPRAC assays in previous publications [9,20,44].

The basic solutions of 7 mM ABTS^+•^ (2,2′-azinobis-3-ethylbenzothiazoline-6-sulfonic acid) and DPPH^•^ (1,1-diphenyl-2-picrylhydrazyl) radicals were prepared in 70% ethanol and diluted approximately 80 times so that the absorption measured was 0.70 (-) at 734 nm (ABTS) or at 517 nm (DPPH).

The ABTS and DDPH methods were used to determine the ability of naringenin and poly(naringenin) to inhibit the free radicals, ABTS^+•^ and DPPH^•^, respectively. The level of ABTS^+•^ or DPPH^•^ inhibition of free radicals was computed according to the equation:

Level of ABTS^+•^ or DPPH^•^ free radical inhibition (%) = [(A_0_ − A_1_)/A_0_] × 100 (1)

where A_0_ is the absorbance of the reference sample and A_1_ is the absorbance of the sample with naringenin or poly(naringenin).

The effect of naringenin or poly(naringenin) on inhibition of ABTS^+•^ and DPPH^•^ radicals is referred to as the Trolox equivalent antioxidant capacity (TEAC). The inhibition level (%) of absorbance was calculated utilizing the standard curve prepared with Trolox (% inhibition level-μM Trolox).

FRAP (Ferric Reducing Antioxidant Power) and CUPRAC (CUPric Reducing Antioxidant Capacity) assays are analogous and are based on the reduction of iron (Fe^3+^→Fe^2+^) and copper ions (Cu^2+^→Cu^1+^), respectively.

The FRAP reaction mixture consisted of acetate buffer (0.3 M, pH 3.6), 10 mM TPTZ (10 mM iron-2,4,6-tripyridyl-S-triazine complex in in 40 mM HCl) and FeCl_3_ (20 mM in water) in a 10:1:1 ratio. Before use, the solution was incubated at 35 °C for 25 min. The CUPRAC solution was made with 0.01 M CuCl_2_, 7.5 × 10^−3^ M neocuproine and ammonium acetate buffer solution (1 M, pH 7.0).

The quantitative ability of naringenin and poly(naringenin) to reduce iron and copper ions was calculated by comparing the change in absorption (ΔA) of the test sample with the value of ΔA determined for the standard solutions without flavonoids. The ΔA value obtained for the samples is directly proportional to the concentration of the antioxidant substance.

### 3.7. Thermal Analysis (Thermogravimetric TG and Differential Scanning Calorimetry DSC)

The thermal stability of naringenin and poly(naringenin) was determined by thermogravimetric analysis (TG). First, 10 mg samples of the powders were placed in alumina crucibles and heated from 25 °C to 800 °C under argon (flow 50 mL/min) at a rate of 5 °C/min (Mettler Toledo Thermobalance, TA Instruments, New Castle, DE, USA).

The temperature ranges of naringenin and poly(naringenin) phase changes were obtained utilizing Differential Scanning Calorimetry (DSC) (Mettler Toledo DSC analyser, TA 2920, TA Instruments). Samples of 5 to 6 mg of flavonoid powders were placed in 100 µL aluminium pans and heated from −80 to 400 °C at a rate of 10 °C/min in air (flow 60 mL/min).

### 3.8. Antimicrobial Properties

Naringenin and (poly)naringenin-laccase powders were suspended in DMSO at concentrations of 1, 5 and 10 mg/mL for the tests. The research used the microorganisms listed in Table 6.

In order to determine the antimicrobial activity by the well diffusion method, the TSA (bacteria) and MEA (yeasts and molds) media were sown with suspensions of appropriate microorganisms in physiological salt, and then the wells were cut out with a sterile cork borer with a diameter of 15 mm. Then 200 µL of suspensions prepared in DMSO were introduced into the wells at the appropriate concentration. After 48 h of incubation at 30 °C, it was noted around which wells a zone of growth inhibition appeared.

## 4. Conclusions

Enzymatic polymerization of naringenin with laccase from Aspergillus Niger or horseradish peroxidase (HRP) allowed oligomeric forms of flavonoid to be obtained. The OH groups (5-OH, 7-OH and 4′-OH) were probably involved in the polymerization reaction; moreover, it was possible to create C-O-C and C-C bonds in oligomeric forms. Polymeric naringenin obtained in the reaction with laccase (poly(naringenin)-laccase) had a higher molecular weight than poly(naringenin)-HRP and, moreover, had a glass transition temperature (66.6 °C) characteristic of polymeric forms. Poly(naringenin)-laccase and poly(naringenin)-HRP had greater thermal stability and oxidation resistance than the reference naringenin. However, poly(naringenin) obtained with laccase, due to its higher molecular weight, had better thermal stability than poly(naringenin)-HRP. 

The activity of poly(naringenin)-lactase against *E. coli* was better at concentrations of 1 and 5 mg/mL. The antimicrobial activity of laccase catalyzed poly(naringenin) against bacteria (*S. aureus*, *B. subtilis*) and fungi (*C. albicans*, *A. niger*) was similar or lower than that of the reference naringenin, which may be due to the limited solubility of poly(flavonoid) and the lower availability of the slowly condensed compound on microorganisms. The antioxidant activity of poly(naringenin) catalyzed by two enzymes in the DPPH method and the ability to reduce Fe^3+^ and Cu^2+^ ions in the FRAP and CUPRAC methods was lower than for the reference naringenin. Some of the naringenin functional groups are lost during the polymerization process, hence the oligomer or polymers may not have higher biological activities than the monomers. However, the ABTS test showed that the antioxidant properties of the polymer forms of naringenin were different than those for the oligomers obtained from laccase and HRP and could depend on the type of enzyme used, the polymerization method and the method of determining the properties.

The precise determination of the structure and properties of the polymeric naringenin was significantly hampered by the limited solubility of the compounds produced in solvents required for the chemical analyses used.

## Figures and Tables

**Table 1:** Characteristics of naringenin, poly(naringenin)-laccase and poly(naringenin)-HRP. The Mn [g/mol] and Mw/Mn were determined by GPC (PS standard).

Sample	M_n_ (g/mol)	M_w_/M_n_
**Naringenin**	below the measuring range	
**Poly(naringenin)-laccase**	4.584 × 10^3^	1.040
**Poly(naringenin)-HRP**	9.972 × 10^2^	1.392

**Table 2:** The activity of naringenin, poly(naringenin)-laccase and poly(naringenin)-HRP to scavenge ABTS^+•^ and DPPH^•^ free radicals, and to reduce iron (FRAP) and copper (CUPRAC) ions.

Method	Parameter	Naringenin	Poly(Naringenin)-Laccase	Poly(Naringenin)-HRP
**ABTS**	Inhibition ABTS^+•^ (%)	55.0 ± 0.22	85.7 ± 0.28	30.4 ± 0.16
	TEAC (mmolT/100 g)	28.4 ± 0.12	93.5 ± 0.31	33.1 ± 0.17
**DPPH**	Inhibition DPPH^•^ (%)	1.0 ± 0.01	no activity	0.2 ± 0.01
	TEAC (mmolT/100 g)	0.9 ± 0.02	no activity	0.2 ± 0.02
**FRAP**	ΔA (-)	0.05 ± 0.002	0.03 ± 0.002	0.02 ± 0.001
**CUPRAC**	ΔA (-)	1.9 ± 0.11	0.402 ± 0.002	no activity

**Table 3:** Values of T_10_, T_20_, T_30_, T_50_, T_55_ and T_60_ of naringenin, poly(naringenin)-laccase and poly(naringenin)-HRP.

	T_10_	T_20_	T_30_	T_50_	T_55_	T_60_
**Naringenin**	307	322	337	436	518	591
**Poly(naringenin)-laccase**	213	356	800	-	-	-
**Poly(naringenin)-HRP**	173	302	365	593	772	-

**Table 4:** DSC analysis of naringenin poly(naringenin)-laccase and poly(naringenin)-HRP.

Sample	T_g_ (°C)	ΔH_m_ (J/g)	T_m_ (°C)	ΔH_o_ (J/g)	T_o_ (°C)
Naringenin	-	164.3	252.9	88.4	281.3 (initial) 330.5 (final)
Poly(naringenin)-laccase	66.6	162.4	182.9	182.2	295.0 (initial) 358.7 (final)
Poly(naringenin)-HRP	-	220.7	25.3	679.5	299.7 (initial) 354.1 (final)

T_g_—glass transition temperature, ΔH_m_—melting enthalpy, T_m_—melting point, ΔH_o_—oxidation and degradation enthalpy, T_o_—oxidation and degradation temperature (initial and final).

**Table 5:** Antimicrobial activity of naringenin and poly(naringenin)-laccase.

Sample	Concentration (mg/mL)	*Escherichia coli*	*Staphylococus aureus*	*Bacillus subtilis*	*Aspergillus niger*	*Candida albicans*
		Growth Inhibition Zone (mm)				
Naringenin	1	0.0 ± 0.0	18.1 ± 0.6 *	23.0 ± 2.0	30.0 ± 0.6	22.3 ± 1.5
	5	0.0 ± 0.0	22.0 ± 1.0	33.0 ± 1.0	32.0 ± 1.0	24.0 ± 1.7
	10	22.0 ± 1.0	30.0 ± 2.0	34.0 ± 1.7	35.0 ± 1.0	24.3 ± 0.6
Poly(naringenin)-laccase	1	20.0 ± 1.0 *	0.0 ± 0.0	22.0 ± 0.0 *	28.0 ± 1.0	26.3 ± 0.6
	5	20.3 ± 0.6	0.0 ± 0.0	23.3 ± 0.6	28.0 ± 0.0	26.0 ± 1.0
	10	22.0 ± 1.0	18.3 ± 0.6	25.0 ± 1.0	30.3 ± 0.6	27.7 ± 0.6

* Secondary growth.

**Table 6:** Biological materials.

Microorganisms		Species	Collection Number
Bacteria		*Escherichia coli*	ATCC 10536
		*Staphylococcus aureus*	ATCC 6538
		*Bacillus subtilis*	NCAIM 01644
Fungus	Yeast	*Candida albicans*	ATCC 10231
	Molds	*Aspergillus niger*	ATCC 16404

ATCC—American Type Culture Collection; NACIM—National Collection of Agricultural and Industrial Microorganisms.

## Data Availability

Not applicable.

## Sample Availability

Samples of the compounds are available from the authors.
